# SIRT1 Prevents Ferroptosis in Corneal Epithelial Cells by Enhancing HIF1α Protein Stability in Dry Eye Disease

**DOI:** 10.1002/advs.202522806

**Published:** 2026-02-25

**Authors:** Lili Lian, Zhenmin Le, Xuanqiao Ye, Zimo Wang, Jiuxiao Li, Chenyu Dong, Yetao Shen, Jiazheng Li, Yueping Ren, Xiaoyin Ma, Wei Chen, Qinxiang Zheng

**Affiliations:** ^1^ Eye Hospital and School of Ophthalmology and Optometry Wenzhou Medical University Wenzhou China; ^2^ Department of Ophthalmology Sir Run Run Shaw Hospital Zhejiang University School of Medicine Hangzhou China; ^3^ Ningde Municipal Hospital Ningde China

**Keywords:** corneal epithelial cells, dry eye disease, ferroptosis, GPX4, SIRT1

## Abstract

Hyperosmotic stress induced by tear film instability significantly exacerbates oxidative damage in corneal epithelial cells, contributing to the pathogenesis of dry eye disease (DED). Ferroptosis, an iron‐dependent form of cell death driven by lipid peroxidation, has been identified as a critical downstream mechanism of oxidative damage in DED. However, its precise regulatory mechanisms remain unclear. In this study, we demonstrate that hyperosmotic stress promotes ferroptosis in corneal epithelial cells by downregulating the NAD‐dependent deacetylase sirtuin 1 (SIRT1). SIRT1 positively regulates GPX4, a pivotal mediator of ferroptosis. Pharmacological activation of SIRT1 using SRT1720 alleviated oxidative damage and suppressed ferroptosis in corneal epithelial cells both in vitro and in vivo. Mechanistically, we further observed that SIRT1 deacetylates the HIF1α, stabilizing it via the ubiquitin‐proteasome pathway. The SIRT1–HIF1α axis positively regulates GPX4 levels, thereby inhibiting ferroptosis activation. These results reveal a previously unrecognized pathway of ferroptosis regulation in DED and suggest a potential therapeutic strategy for reducing oxidative damage in corneal epithelium.

## Graphical abstract

Hyperosmotic stress drives ferroptosis in corneal epithelium via SIRT1 downregulation. This work demonstrates that SIRT1 activation stabilizes HIF1α, which transcriptionally upregulates GPX4 to inhibit lipid peroxidation and cell death. The identified SIRT1/HIF1α/GPX4 axis reveals a novel defense mechanism and potential therapy for dry eye disease.

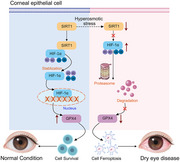

## 1 Introduction

Dry eye disease is a prevalent ocular surface disorder that significantly affects visual health and quality of life, imposing a substantial socio‐economic burden worldwide. Epidemiological data highlight its widespread impact, with a 2021 study estimating a global prevalence of 11.59% [1]. The burden is even more pronounced in Asia, where rates reach approximately 20.1%, notably affecting 21.7% of females and 16.4% of males [2]. Despite its high prevalence, current clinical treatments offer limited effectiveness, and many patients experience chronic symptoms that severely impair their daily lives [3]. The International Dry Eye Workshop II (DEWS II) defines DED as a multifactorial disease. It causes tear film instability and hyperosmolarity, leading to ocular surface inflammation, often accompanied by neurosensory abnormalities [4, 5, 6]. This pathology stems significantly from hyperosmotic stress‐induced injury to corneal epithelial cells, highlighting the critical need for interventions that can counteract this damage. The shortcomings of prevailing clinical management thus reveal a clear gap, necessitating the discovery of new therapeutic targets.

Oxidative stress is widely recognized as a key driver of DED pathology, with increased oxidative damage and diminished antioxidant defenses documented in both patient samples and DED animal models [7]. This imbalance triggers the activation of inflammasomes, which further perpetuate inflammation in a feedback loop [8, 9, 10]. Despite efforts to treat DED with antioxidant therapies, clinical success has been limited, likely due to the complexity of oxidative stress pathways [11]. Among cellular antioxidant systems, glutathione peroxidase 4 (GPX4) plays a critical role in maintaining redox balance by preventing lipid peroxidation [12]. Decreased GPX4 activity can lead to ferroptosis, a regulated form of cell death driven by iron‐dependent lipid peroxidation, distinct from apoptosis and necrosis [13]. Ferroptosis further triggers the release of damage‐associated molecular patterns (DAMPs), which promote immune cells infiltration and lead to sustained inflammation [14].

Ferroptosis research has rapidly advanced in recent years, demonstrating that iron accumulation and reactive oxygen species (ROS) overproduction are central to its pathogenesis [15]. Previous studies have identified diminished lactoferrin levels in DED patients with Sjogren's syndrome, suggesting increased free iron in the ocular microenvironment and thus a predisposition toward ferroptosis [16]. Transcriptomic analyses of DED models have demonstrated downregulated GPX4 expression and activation of ferroptosis pathways in corneal epithelial cells. Notably, aldose reductase AKR1C1 shown to inhibit DED ferroptosis in corneal epithelial cells via nuclear factor erythroid 2‐related factor 2 (NRF2) signaling [17]. Furthermore, liposomes modified with sialic acid‐targeted peptides, loaded with cyclosporine A and the ferroptosis inhibitor Fer‐1, have been shown to alleviate dry eye symptoms by inhibiting the p53‐SLC7A11‐GSH‐dependent ferroptosis process [18]. This targeted ferroptosis intervention strategy offers a promising approach to delay or even block the pathological progression of dry eye and holds potential for future clinical application.

The Sirtuin family consists of seven NAD**^+^**‐dependent deacetylases (SIRT1‐SIRT7), which primarily function by deacetylating target proteins to regulate essential biological processes. SIRT1 is particularly known for its role in cellular metabolism, aging, and stress response [19, 20]. In this study, using both in vivo and in vitro models of dry eye disease, we observed a down‐regulation of SIRT1 expression in corneal epithelial cells undergoing ferroptosis. SIRT1 plays a critical role in ferroptosis regulation and protects against oxidative stress in various diseases. For instance, in renal calcium oxalate nephropathy, SIRT1 inhibits ferroptosis in proximal tubular cells by activating PGC1α, which enhances mitochondrial function and reduces oxidative damage [21]. In neurodegenerative models such as Friedreich's ataxia, SIRT1 modulates the LKB1/AMPK and KEAP1/GSK3β pathways, boosting NRF2‐mediated antioxidant defenses essential for neuronal protection [22]. Similarly, SIRT1 counters α‐synuclein‐induced ferroptosis via the NRF2/HO‐1 and GPX4 pathways in Parkinson's disease models [23]. SIRT1's activation of PGC1α, NRF2, HO‐1, and GPX4 underscores its upstream regulatory role in combating ferroptosis‐related disease.

SIRT1 is highly expressed in corneal epithelial cells and facilitate corneal epithelial repair by modulating cellular migration and the cell cycle [24]. Previous studies on DED have shown that hyperosmotic stress downregulated SIRT1 expression, while nicotinamide mononucleotide (NMN) has been found to reduce IL‐17a expression by enhancing SIRT1 activity [25]. However, the specific impact of SIRT1 on DED‐related oxidative damage and its regulation of ferroptosis in corneal epithelial cells remain unclear. This study investigates the hypothesis that SIRT1 regulates GPX4 expression and mitigates ferroptosis in corneal epithelial cells. We also explore the potential involvement of HIF1α, a known ferroptosis regulator in diverse pathologies. HIF1α is implicated in immune response modulation in the lacrimal gland under DED conditions [26] and may serve as a mediator in the SIRT1‐GPX4 regulatory axis [27]. This investigation provides insights into the SIRT1/HIF1α/GPX4 pathway and its potential as a therapeutic target for inhibiting ferroptosis and alleviating DED pathology in corneal epithelial cells.

## 2 Results

### 2.1 Hyperosmotic‐Induced Ferroptosis in Corneal Epithelial Cells Involves SIRT1 Suppression

**FIGURE 1:**
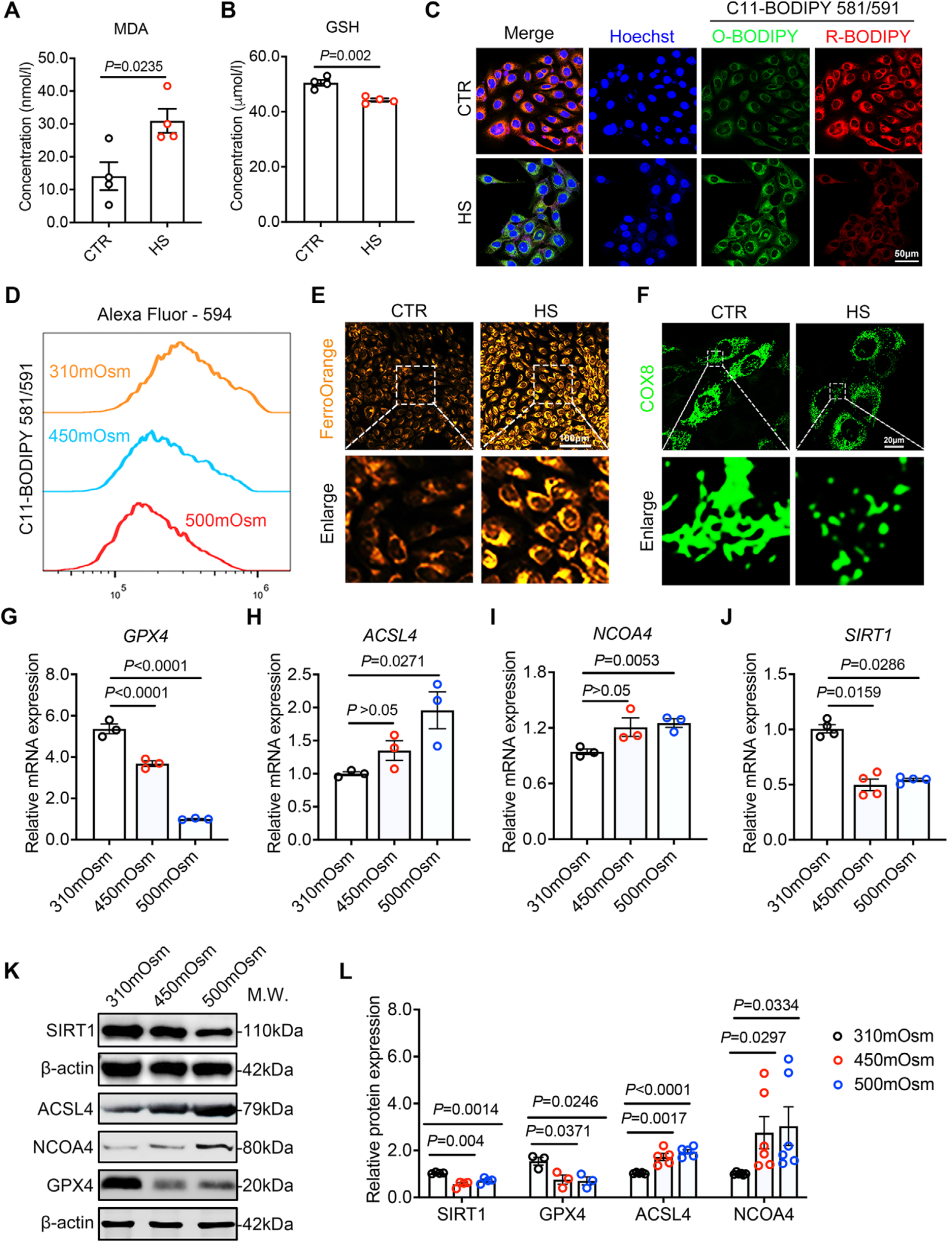
Hyperosmotic stress induces ferroptosis and downregulation of SIRT1 expression in HCECs. (A) Intracellular malondialdehyde concentration was detected in HCECs from the control group (CTR, 310 mOsm, n = 4) and hyperosmotic group (HS, 450 mOsm, n = 4). MDA: malondialdehyde. (B) Detection of intracellular reduced glutathione concentration in the HCECs from the control group (CTR, 310 mOsm, n = 4) and hyperosmotic group (HS, 450 mOsm, n = 4). GSH: reduced glutathione. (C) Representative fluorescence microscopy imaging results of C11‐BODIPY 581/591 dye to detect lipid peroxidation levels in HCECs from the control group and hyperosmotic group. Green indicates oxidized C11‐BODIPY (O‐BODIPY, 510 nm), red indicates reduced C11‐BODIPY (R‐BODIPY, 591 nm), and blue indicates Hoechst. Scale bar: 50 µm. (D) Flow cytometry results of C11‐BODIPY 581/591 dye to detect the level of cell lipid peroxidation in HCECs with or without hyperosmotic stress treated for 24 h. (E) Representative fluorescence microscopy imaging results of FerroOrange probe to detect intracellular Fe^2+^ content in HCECs from the control group and hyperosmotic stress group. Scale bar: 100 µm. (F) The CMV‐COX8‐GFP carrier lentivirus was constructed using inner mitochondrial membrane protein COX8 and transfected into HCECs. IF of COX8 was detected by confocal microscope imaging to observe the morphology of cell mitochondria. Scale bar: 20 µm. (G–J) qRT–PCR analysis of *GPX4* (n = 3), *ACSL4*(n = 3), *NCOA4*(n = 3) and *SIRT1*(n = 4) expression in HCECs with or without hyperosmotic stress treated for 12 h. (K,L) WB analysis of SIRT1(n = 4), GPX4(n = 3), ACSL4(n = 5) and NCOA4(n = 6) expression in HCECs with or without hyperosmotic stress treated for 24 h. The ratio of protein level to β‐actin protein level was used to represent the relative protein intensity. The data are shown as mean ± SEM. Statistical significance was determined as indicated.

To explore the upstream regulatory genes of ferroptosis in DED, we exposed human corneal epithelial cells (HCECs) to hyperosmotic medium (450 mOsm and 500 mOsm) for 24 h. Physiological osmotic pressure (310 mOsm) was designated as the control group. Ferroptosis is typically characterized by lipid peroxidation and iron accumulation [15]. In our study, hyperosmotic stress resulted in a significant increase in the lipid peroxidation product malondialdehyde (MDA) and a decrease in the content of the antioxidant glutathione (GSH) (Figure 1A,B). Lipid oxidation was further visualized using the C11‐BODIPY 581/591 dye. Both immunofluorescence microscopy (Figure 1C) and flow cytometry (Figure 1D) demonstrated a fluorescence shift from red (591 nm, reduced) to green (510 nm, oxidized). To assess iron accumulation, we used the FerroOrange fluorescent probe, which revealed a substantial increase in intracellular Fe^2^^+^ levels under hyperosmotic conditions (Figure 1E). COX8‐labeled fluorescence imaging revealed mitochondrial morphological alterations characteristic of ferroptosis in the hyperosmotic group, including a marked reduction in mitochondrial volume (Figure 1F). Altogether, these findings unequivocally demonstrate that hyperosmotic stress induces ferroptosis in HCECs.

We subsequently evaluated the expression of ferroptosis‐associated genes using qRT‐PCR. In the hyperosmotic stress group, *GPX4* expression was markedly downregulated, whereas *ACSL4* and *NCOA4* were significantly upregulated (Figure 1G–I), consistent with findings from previous studies [17]. Additionally, our analysis revealed a significant downregulation of *SIRT1* in the hyperosmotic stress group (Figure 1J). These transcriptional changes were further corroborated at the protein level (Figure 1K,L). The downregulation of SIRT1 is consistent with the activation of ferroptosis in corneal epithelial cells under dry eye conditions, suggesting a potential regulatory role of SIRT1 in this process.

### 2.2 Downregulation of SIRT1 in Corneal Epithelium of Mice With Dry Eye Disease

**FIGURE 2:**
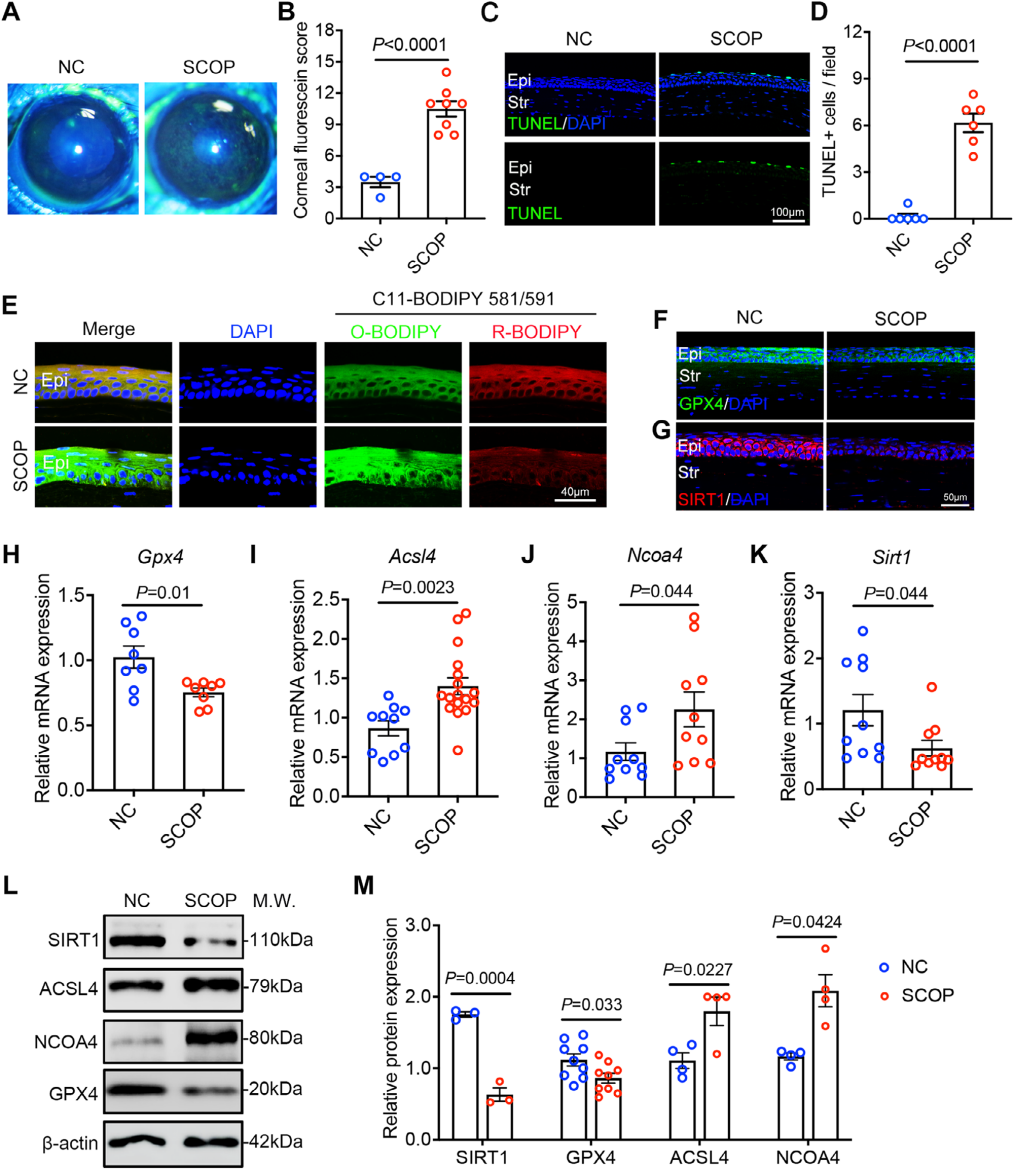
Reduced SIRT1 expression in corneal epithelial cells in a mouse dry eye model. The experimental dry eye mouse model was induced by subcutaneously injecting 0.5 mg/0.2 mL of scopolamine into the groin area three times a day for 5 days. (A,B) Representative corneal fluorescence staining image and quantification of the corneal staining score in C57BL/6J mice injected with SCOP (n = 8) and the control mice(n = 4). (C,D) Representative fluorescence image and quantitative data for TUNEL apoptosis assay to detect apoptotic corneal epithelial cell in C57BL/6J mice injected with SCOP (n = 6) and the control mice (n = 6). Epi: epithelium; Str: stroma. Scale bar: 100 µm. (E) Representative fluorescence microscopy imaging results of C11‐BODIPY 581/591 dye to detect lipid peroxidation levels in frozen corneal sections from SCOP‐induced dry eye mice and control mice. Green indicates oxidized C11‐BODIPY (O‐BODIPY, 510 nm), red indicates reduced C11‐BODIPY (R‐BODIPY, 591 nm), and blue indicates DAPI. Epi: epithelium. Scale bar: 40 µm. (F,G) Representative images of SIRT1 and GPX4 IF staining on frozen corneal sections on SCOP‐induced dry eye mice and control mice. Epi: epithelium; Str: stroma. Scale bar: 50 µm. (H–K) qRT–PCR analysis of *Gpx4*(n = 8), *Acsl4* (n = 10), *Ncoa4*(n = 10) and *Sirt1*(n = 10) expression in cornea from C57BL/6J mice with or without SCOP injected for 5 days. (L,M) WB analysis of SIRT1(n = 3), GPX4(n = 9), ACSL4(n = 4) and NCOA4(n = 4) expression in cornea from C57BL/6J mice with or without SCOP injected for 5 days. The ratio of protein level to β‐actin protein level was used to represent the relative protein intensity. The data are shown as mean ± SEM. Statistical significance was determined as indicated.

To investigate oxidative damage induced by ferroptosis and associated gene expression changes in vivo in DED, we established a dry eye mouse model. Specifically, C57BL/6J mice received subcutaneous injections of scopolamine hydrobromide (SCOP) in the groin region three times daily for five consecutive days. The mice with no treatment were designated as controls. SCOP‐treated mice exhibited pronounced dry eye phenotypes, characterized by more severe corneal epithelial defects and increased apoptosis of corneal epithelial cells compared to controls (Figure 2A–D). To assess ferroptosis, fluorescence staining of corneal sections using the C11‐BODIPY dye revealed elevated lipid peroxidation levels in the corneal epithelium of dry eye mice (Figure 2E). The immunofluorescence results further indicated that the expressions of ferroptosis downstream protein GPX4 and deacetylase SIRT1 in the corneal epithelial of dry eye mice were significantly downregulated (Figure 2F,G). Further, we conducted a comparative analysis of gene expression in corneal tissues of SCOP‐induced dry eye mice and control mice. The expression of ferroptosis‐related genes *Gpx4*, *Acsl4*, and *Ncoa4* shown by qRT‐PCR and WB in dry eye mice were consistent with the results of in vitro cell experiments, as well as *Sirt1* (Figure 2H–M). Collectively, these results demonstrate that SIRT1, a known antioxidant gene, is consistently downregulated in both in vitro and in vivo models of DED, supporting its potential role as a negative regulator of ferroptosis in corneal epithelial cells.

### 2.3 SIRT1 Negatively Regulates Ferroptosis in Corneal Epithelial Cells

**FIGURE 3:**
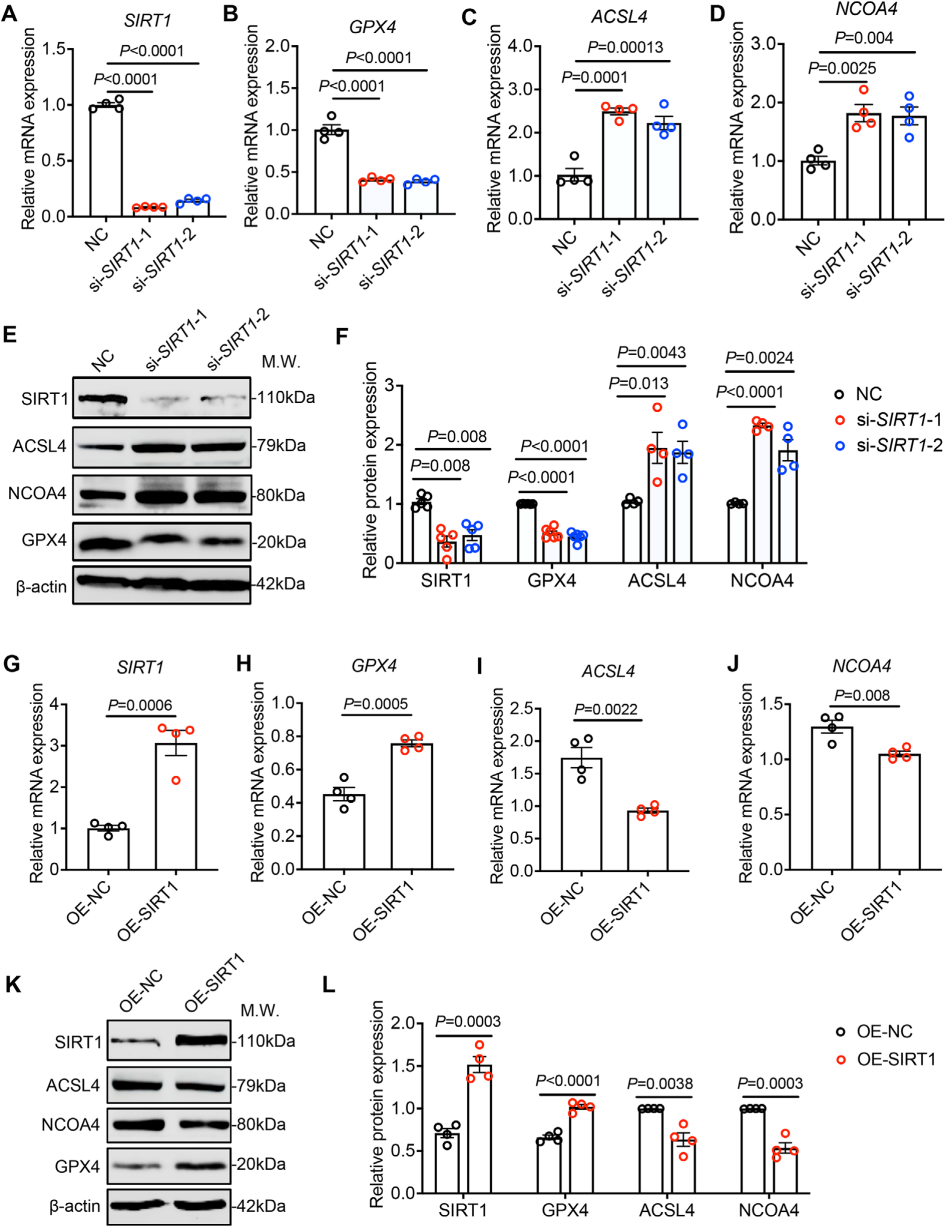
SIRT1 negatively regulates ferroptosis in HCECs. (A–D) qRT‐PCR analysis of *SIRT1*(n = 4), *GPX4*(n = 4), *ACSL4* (n = 4) and *NCOA4*(n = 4) in HCECs transfected with two SIRT1‐specific small interfering RNA fragments and corresponding nonsense control sequences for 48 h, respectively. NC, meaningless interference fragment control; si‐SIRT1, SIRT1 interference fragment. (E,F) WB analysis was performed to quantify the protein expression levels of SIRT1(n = 5), GPX4(n = 6), ACSL4(n = 4) and NCOA4(n = 4) in HCECs transfected with two SIRT1‐specific small interfering RNA fragments and corresponding nonsense control sequences for 72 h, respectively. The ratio of protein level to β‐actin protein level was used to represent the relative protein intensity. (G–J) qRT‐PCR analysis of *SIRT1*(n = 4), *GPX4*(n = 4), *ACSL4* (n = 4) and *NCOA4*(n = 4) in HCECs with stable overexpression of SIRT1(OE‐SIRT1) and in control HCECs (OE‐NC). K, L) WB analysis was conducted to assess the protein expression levels of SIRT1(n = 4), GPX4(n = 4), ACSL4(n = 4) and NCOA4(n = 4) in HCECs overexpressing SIRT1(OE‐SIRT1) compared to control HCECs (OE‐NC). Relative protein expression levels were normalized to β‐actin. The data are presented as mean ± SEM. Statistical significance was determined as indicated.

Previous studies have demonstrated that SIRT1 alleviates intracellular oxidative stress and enhances lipid metabolism, playing a direct or indirect role in the regulation of ferroptosis [21, 22, 23]. Building on these findings, we next proceeded to explore whether SIRT1 regulates the ferroptosis involved in DED. Small interfering RNA (siRNA) and CMV promoter–driven lentiviral vectors were used to achieve knockdown and overexpression of SIRT1 in HCECs. The efficiency of SIRT1 modulation was confirmed by qRT‐PCR (Figure 3A,G) and WB (Figure 3E,K). We subsequently investigated the regulatory impact of SIRT1 on ferroptosis marker GPX4, as well as the intermediary regulators ACSL4 and NCOA4. In SIRT1‐knockdown HCECs, qRT‐PCR analysis revealed a marked decrease in GPX4 mRNA (Figure 3B) and protein expression (Figure 3E), accompanied by a significant upregulation of ACSL4 (Figure 3C,E) and NCOA4 (Figure 3D,E). These expression patterns closely mirrored those observed in the corneal epithelium cells of DED models indicated. Conversely, overexpression of SIRT1 in HCECs resulted in elevated GPX4 mRNA (Figure 3H) and protein levels (Figure 3K), along with suppressed expression of ACSL4 (Figure 3I,K) and NCOA4 (Figure 3J,K), indicating the protective effect of SIRT1 in inhibiting ferroptosis. These results show that SIRT1 downregulation may be a critical molecular event driving ferroptosis in hyperosmolarity‐induced corneal epithelial cells. Moreover, therapeutic activation of SIRT1 could represent a potential strategy for mitigating ferroptosis in the context of DED.

### 2.4 SRT1720 Alleviates Hyperosmotic‐Induced Ferroptosis in Corneal Epithelial Cells

**FIGURE 4:**
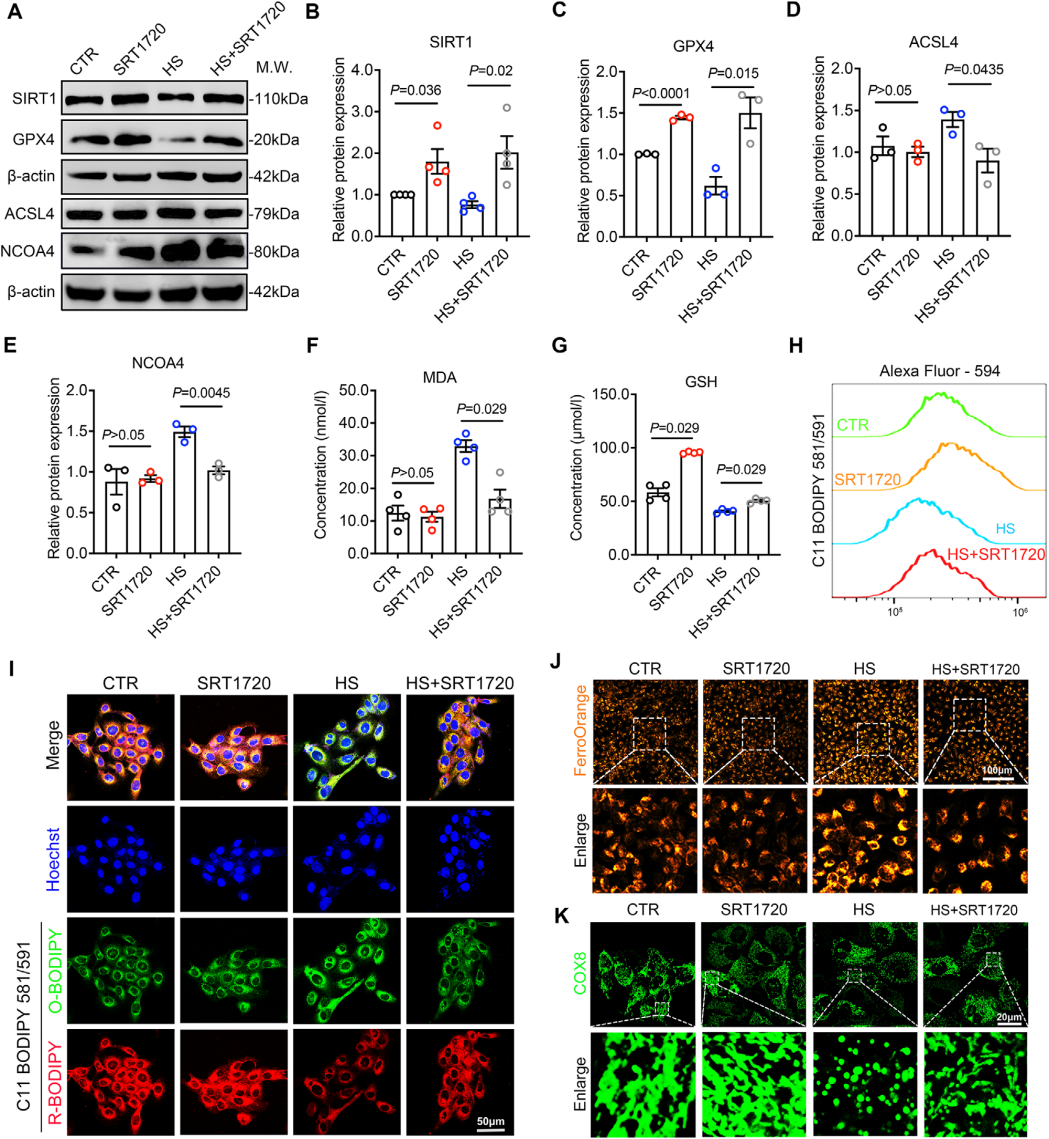
The SIRT1 agonist SRT1720 inhibits hyperosmotic‐induced ferroptosis in HCECs. (A–E) HCECs were pretreated with 1 µm SRT1720 for 2 h prior to exposure to 450 mOsm hyperosmotic stress for 24 h. WB was performed to quantitatively assess the protein expression levels of SIRT1, GPX4, ACSL4, and NCOA4 in the control group (CTR), SRT1720 treatment group (SRT1720), hyperosmotic group (HS), and SRT1720 pretreated hyperosmotic group (HS+SRT1720). Quantitative analysis of protein expression levels of SIRT1(n = 4), GPX4(n = 3), ACSL4(n = 3) and NCOA4(n = 3), normalized to β‐actin. (F) Intracellular malondialdehyde concentration was detected in HCECs from the control group (CTR, n = 4), SRT1720 treatment group (SRT1720, n = 4), hyperosmotic group (HS, n = 4) and SRT1720 pretreated hyperosmotic group (HS+SRT1720, n = 4). MDA: malondialdehyde. (G) Detection of intracellular reduced glutathione concentration in the HCECs from the control group (CTR, n = 4), SRT1720 treatment group (SRT1720, n = 4), hyperosmotic group (HS, n = 4) and SRT1720 pretreated hyperosmotic group (HS+SRT1720, n = 4). GSH: reduced glutathione. (H) Flow cytometry analysis using C11‐BODIPY 581/591 dye was conducted to assess lipid peroxidation levels. (I)Representative fluorescence microscopy imaging results of C11‐BODIPY 581/591 dye to detect lipid peroxidation levels in HCECs. Green indicates oxidized C11‐BODIPY (O‐BODIPY, 510 nm), red indicates reduced C11‐BODIPY (R‐BODIPY, 591 nm), and blue indicates Hoechst. Scale bar: 50 µm. (J) Representative fluorescence microscopy imaging results of FerroOrange probe to detect intracellular Fe^2+^ content in HCECs. Scale bar: 100 µm. (K) IF of COX8 was detected by confocal microscope imaging to observe the morphology of cell mitochondria. Scale bar: 20 µm. Data are presented as mean ± SEM from at least three independent experiments. Statistical significance was determined as indicated.

After initially investigating the regulatory role of SIRT1 in ferroptosis of corneal epithelial cells, we further explored whether SIRT1 activation could prevent hyperosmolarity‐induced ferroptosis in HCECs. To this end, we selectively activated SIRT1 using SRT1720. HCECs were pretreated with 1 µm SRT1720 for 2 h prior to the induction of ferroptosis by hyperosmotic stimulation. Western blot analysis confirmed the activation of SIRT1 at the protein level (Figure 4A,B). At the molecular level, SRT1720 treatment reversed the expression changes of ferroptosis‐related proteins induced by hyperosmotic stress. Specifically, it upregulated the expression of GPX4 while downregulating ACSL4 and NCOA4 (Figure 4A–E). In addition, biochemical assays showed that SRT1720 significantly reduced levels of the lipid peroxidation product MDA and increased levels of antioxidant GSH in hyperosmotic‐stressed HCECs (Figure 4F,G). To further assess lipid peroxidation, we performed flow cytometry and immunofluorescence using the C11‐BODIPY 581/591 dye. In SRT1720‐treated hyperosmotic HCECs, a rightward shift in the emission wavelength of reduced C11‐BODIPY was observed (Figure 4H), which was further confirmed by immunofluorescence imaging (Figure 4I). Live‐cell imaging using the FerroOrange fluorescent probe demonstrated that SRT1720 inhibited Fe2^+^ accumulation in HCECs under hyperosmotic stress (Figure 4J). Moreover, COX‐8 labeled fluorescence imaging revealed that mitochondrial morphology was better preserved in the SRT1720‐treated hyperosmotic group compared to the hyperosmotic group (Figure 4K). These results illustrated that activation of SIRT1 by SRT1720 can mitigate hyperosmolarity‐induced ferroptosis in corneal epithelial cells.

### 2.5 SIRT1‐Mediated Inhibition of Ferroptosis Alleviates Corneal Epithelial Injury in DED Mice

**FIGURE 5:**
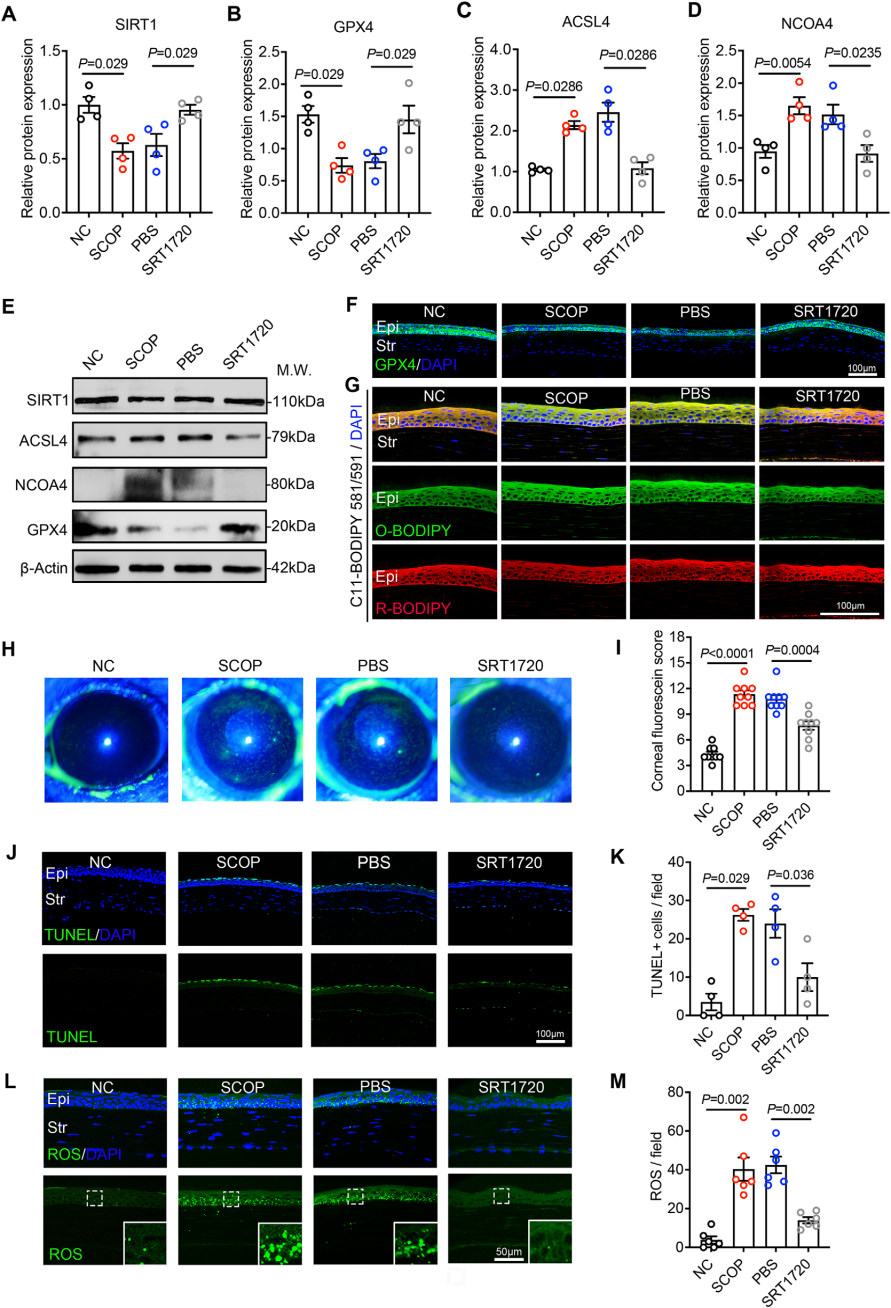
SRT1720 inhibits ferroptosis related injury in dry eye corneal epithelial cells. SCOP induced dry eye mouse model, and the SIRT1‐specific agonist SRT1720 eye drops was applied to the ocular surface of mice three times daily for five consecutive days. (A–E) WB analysis was conducted to assess the expression of SIRT1 (n = 4), GPX4 (n = 4), ACSL4 (n = 4), and NCOA4 (n = 4) in the corneas of control group mice (NC), SCOP‐induced dry eye mice (SCOP), PBS treated dry eye mice (PBS), and SRT1720 treated dry eye mice (SRT1720). The ratio of protein level to β‐actin protein level was used to represent the relative protein intensity. (F) Representative images of GPX4 IF staining on frozen corneal sections. Epi: epithelium; Str: stroma. Scale bar: 100 µm. (G) Representative fluorescence microscopy imaging results of C11‐BODIPY 581/591 dye to detect lipid peroxidation levels on frozen corneal sections. Green indicates oxidized C11‐BODIPY (O‐BODIPY, 510 nm), red indicates reduced C11‐BODIPY (R‐BODIPY, 591 nm), and blue indicates DAPI. Scale bar: 100 µm. (H,I) Representative corneal fluorescence staining image and quantification of the mice corneal staining score (n = 9 per group). (J,K) Representative fluorescence image and quantitative data for TUNEL apoptosis assay to detect apoptotic corneal epithelial cell in experimental mice (n = 4 per group). Epi: epithelium; Str: stroma. Scale bar: 100 µm. (L,M) CM‐H2DCFDA fluorescent probe was used to label intracellular ROS accumulation and ROS count statistical results were obtained (n = 6 per group). Epi, corneal epithelium; Str, Corneal stroma. Scale bar: 50 µm.Data are presented as mean ± SEM from at least three independent experiments. Statistical significance was determined as indicated.

To explore whether blocking SIRT1‐associated ferroptosis signaling axis can protect corneal epithelial cells from oxidative damage in DED, we conducted in vivo studies using the ferroptosis inhibitor deferoxamine (DFO) and the SIRT1 agonist SRT1720, respectively. The DED model was established by continuously administering scopolamine to C57BL/6J mice, and at the same time, DFO or SRT1720 eye drops were given for intervention treatment. Mice that received no treatment served as the control group, while those that received PBS eye drops were designated as the negative control group. DFO is an iron‐chelating agent that reduces cellular iron uptake and limits ROS production [28]. Our results demonstrated that GPX4 expression was significantly downregulated in the corneal tissues of SCOP‐induced dry eye mice, irrespective of PBS treatment. DFO can upregulate the expression of GPX4 (Figure S1A,B), exerting an anti‐ferroptosis effect and protecting corneal epithelial cells from oxidative damage (Figure S1C–H). These results support the use of Fer‐1, another ferroptosis inhibitor, in the previous study to treat corneal epithelial cell damage in DED [17]. Notably, SRT1720 not only increased GPX4 expression but also suppressed hyperosmotic‐induced upregulation of NCOA4 and ACSL4 (Figure 5A–F). C11‐BODIPY fluorescent probe showed that SRT1720 eye drops could significantly inhibit the level of lipid oxidation in the corneal epithelial cell layer of dry eye mice (Figure 5G). SRT1720 demonstrated significant therapeutic efficacy as a treatment for dry eye, effectively suppressing oxidative damage and apoptosis in corneal epithelial cells (Figure 5H–M). Consistent with our hypothesis, these findings further demonstrate SIRT1, as a key upstream regulatory gene in inhibiting ferroptosis, plays a significant protective function against oxidative damage in DED corneal epithelial cells

### 2.6 HIF1α Mediated the Positive Regulation of SIRT1 on GPX4

**FIGURE 6:**
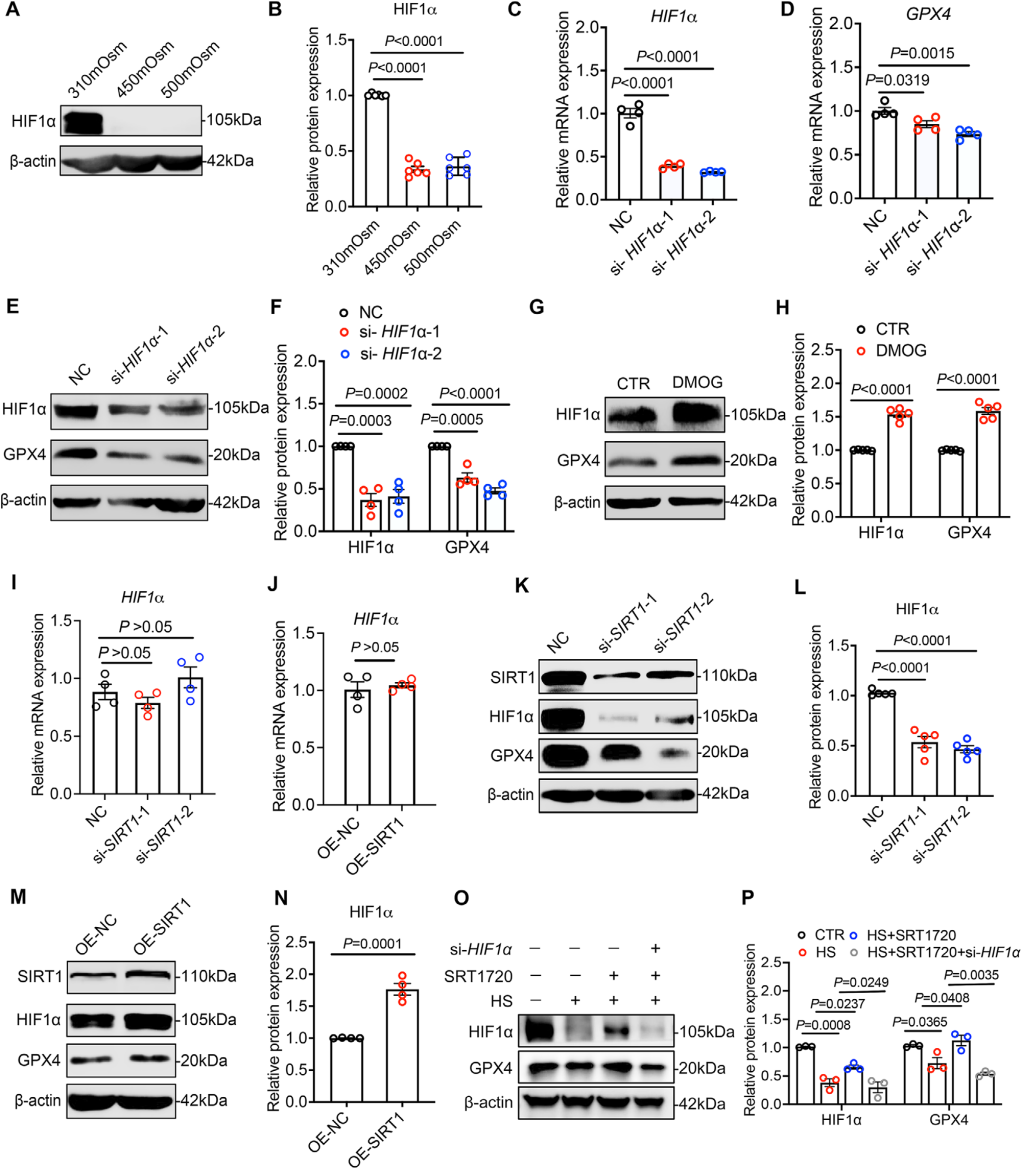
HIF1α mediates the regulation of GPX4 by SIRT1 as an intermediate molecule. (A,B) WB analysis of HIF1α (n = 6) expression in HCECs with or without 450mOsm hyperosmotic stress treated for 24 h. Relative protein expression levels were normalized to β‐actin. (C,D) qRT‐PCR analysis of *HIF1α* (n = 4) and *GPX4* (n = 4) in HCECs transfected with two Hif1α‐specific small interfering RNA fragments (si‐*HIF1α*‐1) and corresponding nonsense control sequences (NC) for 48 h. (E,F) WB analysis was performed to quantify the protein expression levels of HIF1α(n = 4) and GPX4(n = 4) in HCECs transfected with two HIF1α‐specific siRNAs or corresponding nonsense control sequences for 72 h. The ratio of protein level to β‐actin protein level was used to represent the relative protein intensity. (G,H) WB analysis was performed to quantify the protein expression levels of HIF1α(n = 5) and GPX4(n = 5) in HCECs with or without 50 µm DMOG treated for 24 h. (I) qRT‐PCR analysis of *HIF1α* (n = 4) in HCECs after transfection with two SIRT1‐specific siRNAs or nonsense control sequences for 48 h, respectively. (J) qRT‐PCR analysis of *HIF1α* (n = 4) in HCECs with stable overexpression of SIRT1(OE‐SIRT1) and in control HCECs (OE‐NC). (K,L) WB analysis was performed to quantify the protein expression levels of SIRT1, HIF1α, and GPX4 in HCECs transfection with two SIRT1‐specific siRNAs for 72 h. The ratio of HIF1α protein level to β‐actin protein level was used to represent the relative protein intensity(n = 5). (M,N) WB analysis was used to assess the protein expression levels of SIRT1, HIF1α, and GPX4 in HCECs overexpressing SIRT1 compared to control HCECs. Relative HIF1α protein expression levels were normalized to β‐actin(n = 4). (O,P) WB analysis of HIF1α and GPX4 protein expression levels in HCECs under the following conditions for 72 h: normal control, hyperosmotic stress (HS, 450 mOsm), HS supplemented with SRT1720 (1 µm), and HS with SRT1720 following HIF1α knockdown (si‐*HIF1α*). Quantification of HIF1α (n = 3) and GPX4 (n = 3) protein expression levels normalized to β‐actin. NC, meaningless interference fragment control; si‐SIRT1, SIRT1 interference fragment; si‐HIF1α, HIF1α interference fragment. Data are presented as mean ± SEM from at least three independent experiments. Statistical significance was determined as indicated.

Next, we sought to further elucidate the molecular mechanisms underlying SIRT1‐mediated regulation of ferroptosis in dry eye corneal epithelial cells. Given the pronounced alterations observed in key ferroptosis‐related genes, including GPX4, ACSL4, and NCOA4, we hypothesized the involvement of a common upstream regulator. Hypoxia‐inducible factor 1‐alpha (HIF1α), a pivotal transcription factor in hypoxic responses, has been implicated in the regulation of several ferroptosis‐associated signaling pathways. Previous studies have demonstrated that HIF1α positively regulates the expression of the upstream protective factor SLC7A11^27^, essential for GPX4 activity, and directly interacts with the promoters of ACSL4 [29] and NCOA4 [30]. Building on these findings, we aimed to investigate whether HIF1α acts as an intermediary in the regulation of ferroptosis by SIRT1 under hypertonic stress conditions. First, western blot analysis revealed a significant downregulation of HIF1α protein expression 24 h after hyperosmotic exposure, suggesting its differential expression in dry eye conditions (Figure 6A,B). To probe the regulatory role of HIF1α, we utilized siRNA to inhibit its expression and the HIF1α stabilizer dimethyloxyglycine (DMOG) to enhance its protein stability. Consistent results from RT‐PCR and western blot analysis confirmed that HIF1α positively regulates GPX4 expression at both the mRNA and protein levels (Figure 6C–H). To determine whether SIRT1 modulates ferroptosis via HIF1α, we conducted SIRT1 knockdown and overexpression experiments. Interestingly, while SIRT1 did not significantly alter HIF1α mRNA levels (Figure 6I,J), it markedly increased HIF1α protein expression (Figure 6K–N). Subsequent western blot (Figure 6O,P) and fluorescence imaging using C11 BODIPY and FerroOrange probes (Figure S2A,B) demonstrated that knockdown of HIF1α abolishes the protective effect of SRT1720 against hyperosmosis‐induced ferroptosis in HCECs. These results suggest that SIRT1 may enhance HIF1α protein expression at the post‐translational level, thereby indirectly inhibiting ferroptosis through HIF1α‐mediated transcriptional regulation.

### 2.7 SIRT1 Deacetylated HIF1α to Increase its Protein Stability

**FIGURE 7:**
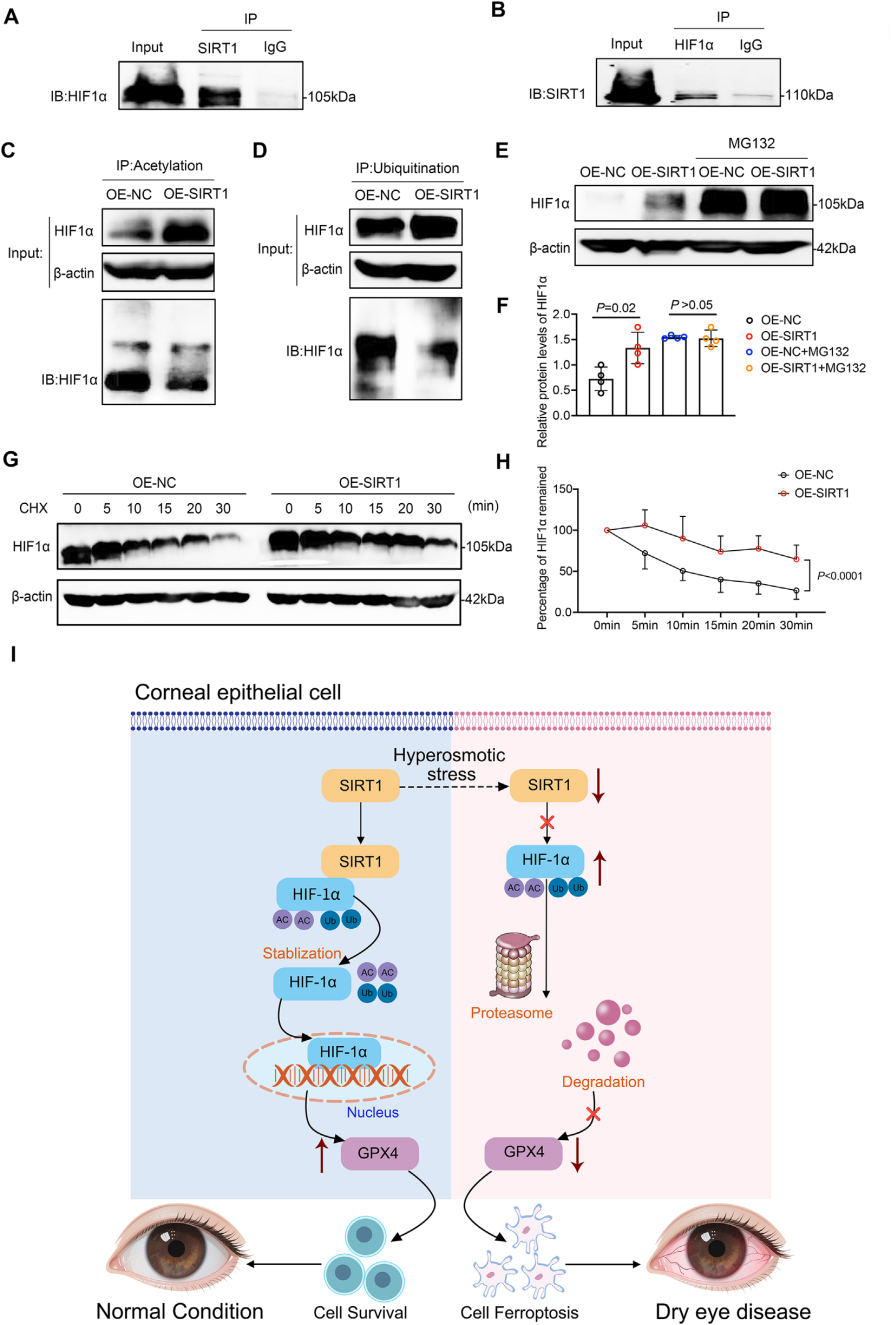
SIRT1 promotes HIF1α protein stability by inhibiting the ubiquitin‐proteasome system. (A,B) Co‐immunoprecipitation assays showing the interaction between SIRT1 and HIF1α. IP: immunoprecipitation, IB: immunoblotting. (C) Acetylation levels of HIF1α in SIRT1‐overexpressing (OE‐SIRT1) and control (OE‐NC) cells were assessed by IP with an acetylation‐specific antibody followed by IB for HIF1α. (D) Ubiquitination levels of HIF1α in OE‐SIRT1 and OE‐NC cells were determined by IP with a ubiquitin‐specific antibody followed by IB for HIF1α. (E) Immunoblot analysis of HIF1α protein levels in OE‐SIRT1 and OE‐NC HCECs treated with or without MG132, a proteasome inhibitor. (F) Quantification of HIF1α protein levels from (E), normalized to β‐actin. (G) Protein stability of HIF1α in OE‐SIRT1 and OE‐NC HCECs was analyzed by cycloheximide (CHX) chase assays at the indicated time points (0–30 min). (H) Quantitative analysis of the percentage of HIF1α remaining over time from the CHX chase assay in (G). (I) A molecular mechanism diagram showing how hyperosmotic stress downregulates SIRT1, targeting HIF1α to induce ferroptosis in corneal epithelial cells. Data are presented as mean ± SEM from at least three independent experiments. Statistical significance was determined as indicated.

Acetylation, a dynamic and reversible post‐translational modification, is regulated by deacetylases. In this study, we investigated the post‐translational regulatory role of the deacetylase SIRT1 on HIF1α in HCECs. Co‐immunoprecipitation assays (Co‐IP) confirmed a direct physical interaction between SIRT1 and HIF1α at the protein level (Figure 7A,B) and further showed that SIRT1 reduced both the acetylation and ubiquitination levels of HIF1α (Figure 7C,D). Previous studies have demonstrated that acetylation promotes ubiquitination and accelerates proteasomal degradation of target proteins [31]. Consistent with this, treatment with the proteasome inhibitor MG132 abolished the SIRT1‐mediated changes in HIF1α protein levels, indicating that the regulatory effect of SIRT1 is proteasome‐dependent (Figure 7E,F). To further assess the impact of SIRT1 on HIF1α protein stability, we measured HIF1α degradation following cycloheximide (CHX) treatment to block new protein synthesis. Compared with the control group, HIF1α degradation was significantly delayed in SIRT1‐overexpressing cells (Figure 7G,H), confirming that SIRT1 stabilizes HIF1α. In conclusion, these findings suggest that SIRT1 enhances the stability of HIF1α by deacetylating the protein and reducing its ubiquitination, thereby preventing proteasomal degradation and promoting HIF1α‐dependent cellular responses.

## 3 Discussion

This study elucidated the regulatory role of SIRT1 in GPX4‐mediated ferroptosis in dry eye corneal epithelial cells. We demonstrated that SIRT1 expression was significantly downregulated in both in vivo and in vitro models of DED. The reduction in SIRT1 expression correlated with an increase in ferroptosis markers, such as lipid peroxidation and iron accumulation, along with decreased GPX4 levels. The pharmacological activation of SIRT1 markedly inhibited ferroptosis in DED‐affected corneal epithelial cells and mitigated cellular damage. Mechanistically, we found that SIRT1 regulated the stability of HIF1α, a key transcription factor in oxidative stress responses, which in turn influenced GPX4 transcription. These findings suggest that SIRT1 functions as a critical modulator of ferroptosis in DED, providing insights into potential therapeutic targets for treating DED‐associated oxidative damage.

Chronic inflammation, oxidative stress, abnormal immune regulation, and corneal epithelial cell defects create a vicious cycle, which has been shown to contribute to the development of dry eye disease [32]. Ferroptosis, triggered by excessive oxidative stress, presents valuable therapeutic potential for breaking the vicious cycle [17, 33]. Our results support the activation of ferroptosis in corneal epithelial cells induced by tear hypertonic. Consistent with previous studies [17], dry eye corneal epithelial cells showed decreased GPX4 and increased ACSL4 expression at the molecular level. It is worth noting that we also observed an upregulation of NCOA4 expression. NCOA4 is a selective receptor for ferritin, mediating ferritin autophagy, and plays a critical role in maintaining intracellular iron homeostasis [34]. Elevated NCOA4 expression may lead to increased intracellular free iron levels, but its regulatory mechanisms in dry eye epithelial cells remain to be clarified. It can be seen that hypertonic induction of corneal epithelial cell ferroptosis involves multiple ferroptosis‐related signaling pathways.

Sirtuins are a family of highly conserved NAD^+^‐dependent histone deacetylases. As a key regulator of ferroptosis, SIRT1 modulates the activity of critical ferroptosis‐related proteins through deacetylation [35]. SIRT1 plays a multifaceted role in regulating iron metabolism and influencing ferroptosis [23, 36]. This regulatory function is particularly important for the treatment of diseases associated with oxidative stress and ferroptosis. Our findings highlight a potential protective mechanism by which SIRT1 can reduce oxidative stress and inhibit ferroptosis, thus preserving cellular integrity in the corneal epithelium. The identification of SIRT1 as a regulatory factor for ferroptosis in corneal epithelial cells under dry eye conditions has significant implications. Previous studies have demonstrated that SIRT1‐mediated deacetylation primarily inhibits ferroptosis via two main pathways: (1) deacetylation of p53, which suppresses the p53‐dependent downregulation of SLC7A11 [37, 38]; and (2) deacetylation of Nrf2, which promotes the upregulation of Nrf2‐mediated GPX4 expression [39, 40]. Interestingly, our experimental results suggest that HIF1α may also serve as a critical downstream target of SIRT1 in ferroptosis. Inhibition of HIF1α expression was found to downregulate GPX4, potentially attenuating oxidative stress associated with ferroptosis. SIRT4, another member of the Sirtuin family, has been shown to modulate HIF1α expression. Notably, SIRT4 can inhibit ferroptosis through the HIF1α/HO‐1 pathway and exert a protective effect in models of acute pancreatitis [41]. However, most current studies on the Sirtuin–HIF1α–ferroptosis axis have focused primarily on phenotypic observations and rescue experiments, with limited investigation into the underlying molecular regulatory mechanisms. Based on our findings that HIF1α may act as an intermediary in SIRT1‐mediated regulation of GPX4 in corneal epithelial cells, we propose that further exploration into the post‐translational modification of HIF1α by SIRT1—particularly its deacetylation—may offer novel mechanistic insights.

The potential mechanism underlying the observed effects of SIRT1 on ferroptosis in DED is the regulation of HIF1α stability. HIF1α is a key transcription factor that responds to hypoxic and oxidative stress by upregulating antioxidant genes, including GPX4. Under DED conditions, the reduced expression of SIRT1 may lead to decreased HIF1α stability, resulting in lower GPX4 transcription and an increased susceptibility to ferroptosis. The activation of SIRT1 may therefore stabilize HIF1α, allowing it to maintain GPX4 levels and prevent ferroptosis. This hypothesis is supported by evidence in other cell types where HIF1α has been shown to upregulate GPX4 in response to oxidative stress, protecting cells from ferroptosis [42, 43]. However, HIF‐1α plays a dual role that varies depending on the severity of cellular hypoxia and the surrounding microenvironment. HIF1α may regulate GPX4 through direct or indirect means, and that future studies could explore the precise transcriptional mechanism. Additionally, given that SIRT1 is a NAD^+^‐dependent enzyme, fluctuations in NAD^+^ levels in the corneal epithelium under DED conditions may also contribute to the dysregulation of SIRT1 and, consequently, HIF1α and GPX4. Future studies could explore these interactions in greater detail to clarify the precise molecular mechanisms involved.

Post‐translational modifications, such as acetylation and ubiquitination, are key regulators of substrate proteins and enzymes. SIRT1, a nicotinamide‐dependent deacetylase, influences protein stability, gene transcription, and metabolism by deacetylating histone and non‐histone lysine residues. Ubiquitination targets proteins for degradation via the proteasome [44]. Our study demonstrates that SIRT1 stabilizes HIF1α by inhibiting its ubiquitin‐proteasome degradation in HECEs. This research not only enhances our understanding of the molecular regulation of ferroptosis in dry eye but also proposes a novel therapeutic approach targeting deacetylation to inhibit ferroptosis. Epigenetic modifications, which regulate cellular functions without altering genetic material, present promising therapeutic possibilities.

Although our research results provide valuable insights into the role of SIRT1 in regulating ferroptosis in DED, there are some limitations in this study. First, in this study, the deacetylation modification sites of SIRT1 regulating HIF1α have not been identified. There is a lack of research on the physiological regulatory effects of specific sites on the ubiquitination modification and protein stability of HIF1α, as well as corresponding verification of in vivo models. Second, this study mainly focuses on the in vitro and animal models of DED, which may not fully capture the complexity of the pathogenesis of human DED. Future research should include clinical studies to verify the therapeutic potential of SIRT1 activators or iron ptosis inhibitors in human patients. Furthermore, exploring the interactions among SIRT1, NAD^+^ metabolism and other cell death pathways in DED can provide a broader understanding of the molecular mechanisms driving corneal epithelial oxidative damage.

## 4 Conclusion

In summary, our results indicate that SIRT1 mediates the ferroptosis and related oxidative damage in dry eye corneal epithelial cells. Further preclinical and clinical studies are crucial for evaluating whether SIRT1‐related ferroptosis inhibitors may provide effective treatment options for DED.

## 5 Experimental Section

### 5.1 Cell Culture and Treatments

The immortalized human corneal epithelial cells (HCE‐T, often referred to as HCE‐2 in the literature, RRID: CVCL_4U66) were purchased from American Type Culture Collection (CRL‐11135; ATCC, USA). Cell line identity was authenticated using Short Tandem Repeat (STR) profiling by an external service provider (Procell Life Science & Technology Co., Ltd., China), which confirmed the cells to be of the HCE‐T lineage. The cells cultured at 37°C and 5% CO_2_ in DMEM/F‐12 medium (Gibco, USA) supplemented with 10% FBS (Gibco, USA) and 1% ITS‐X (Gibco, USA). Hyperosmolar stress models were established by adding 70 mm or 90 mm NaCl (Aladdin, China) to create 450 mOsm and 500 mOm environments, respectively, while 310 mOsm was used as the control. Cells were pretreated with the SIRT1 agonist SRT1720 (1 µm; Topscience, China) 2 h before hyperosmotic stress and with HIF1α agonist DMOG (50 µm; Selleck, USA) for 24 h.

In this study, SRT1720 was applied at a concentration of 1 µm to activate SIRT1. While SRT1720 has been reported to potentially affect other sirtuins (e.g., SIRT2, SIRT3) at higher concentrations (typically ≥10 µm), the use of a lower concentration (1 µm) in our experiments is expected to enhance selectivity for SIRT1 and reduce the likelihood of off‐target effects.

### 5.2 siRNA and Lentiviral Transfection

HCECs were transfected with siRNAs (40 nm; Genechem, China) using Lipojet (SignaGen, USA) or with lentiviral vectors (MOI 100; Genechem, China). Cells stably overexpressing SIRT1 were selected using puromycin (Beyotime, China). siRNA sequences are listed in Table S1.

### 5.3 Animal Model and Treatment

Female C57BL/6J mice (2 months old; Zhejiang Vital River Laboratory Animal Technology, China) were treated per the guidelines of Wenzhou Medical University's Ethics Committee. DED was induced with subcutaneous injections of scopolamine hydrobromide (0.5 g/0.2 mL; Sigma–Aldrich, USA) three times daily for five days. Treatment groups received eye drops of SRT1720 (1 µm) or deferoxamine mesylate salt (DFO; Sigma–Aldrich, USA) dissolved in PBS, while controls received PBS only.

### 5.4 Corneal Epithelial Sodium Fluorescein Staining

Mice were treated with 0.5 mL of 5% sodium fluorescein and observed under cobalt blue light with a slit‐lamp microscope (SLM‐7E; Chongqing Kanghua, China). Staining scores were determined using the NEI grading system.

### 5.5 Quantitative RT‐PCR

HCECs or chopped corneas of mice were lysed by RNeasy Lysis Buffer RLT (QIAGEN, Germany) with 2‐Mercaptoethanol (Sigma–Aldrich, USA), then washed with 70% ethanol, Buffer RW1, Buffer RPE (Sigma–Aldrich, USA), and finally dissolved with DEPC‐Treated Water (Thermo, USA) to obtain total RNA. SYBR qPCR Master Mix (Vazyme, China) and the primers (Tsingke Biotech Co., Ltd., China) are used for Quantitative RT‐PCR on QuantStudio 6 Flex Real‐Time PCR System (Applied Biosystems, Thermo, USA) China). The relative mRNA expression was measured according to the Cycle Threshold of the samples. The sequence of relevant primers are shown in the Table S2.

### 5.6 Western Blot Analysis

Protein was extracted from HCECs or chopped corneas of mice by Radio Immunoprecipitation Assay (RIPA) Lysis Buffer with Phenylmethanesulfonyl fluoride (PMSF) and Protease inhibitor (Beyotime Biotechnology, China). The total protein concentration was determined Detergent Compatible Bradford Protein Assay Kit (Beyotime Biotechnology, China). Each protein sample was diluted with ddH2O and SDS‐PAGE Sample Loading Buffer (Beyotime Biotechnology, China) to the same concentration, and then denatured by heat.

The protein samples are separated with SDS‐PAGE electrophoresis and then transferred to 0.22 µm nitrocellulose transfer memberance (GE HealthCare, USA). The memberance was blocked with 5% no‐fat milk in TBST at room temperature for 2 h and incubated with the primary antibodies at 4°C overnight. The primary antibodies are as follows: anti‐SIRT1 (#ab189494; Abcam, UK), anti‐GPX4 (#ab125066; Abcam, UK), anti‐HIF1α (#ab179483; Abcam, UK) and anti‐β‐actin (#AF5003; Beyotime Biotechnology, China). After being washed, the memberance was incubated at room temperature with Goat Anti‐Rabbit IgG HRP (Biosharp, China) secondary antibody for 1 h. ECL chemiluminescence reagent BeyoECL Moon (Beyotime Biotechnology, China) was added to the memberance in the dark, and the memberance was exposed by the multifunctional imager (Amersham Imager 680; GE HealthCare, USA). The strips were quantitative analysed by using Image J software with β‐actin as the internal control.

### 5.7 Co‐Inmunoprecipitation

Extract the protein of cell samples according to the routine procedures. Take out part of the protein for the input group, and the rest for subsequent IP group. The Protein A/G Magnetic Beads (MedChemExpress, USA) were cleaned with PBST and magnetically separated for several times. Then, 400 µL PBST and 3 µL corresponding antibodies were added to the 40 µL magnetic beads. The primary antibodies are as follows: Anti‐Mouse IgG H&L (#ab205719; Abcam, UK), anti‐Pan Acetylation (#66289‐1‐Ig; Proteintech, China), anti‐Ubiquitin (#3936S; Cell Signaling Technology, USA), anti‐SIRT1, and anti‐HIF1α. After 2–4 h of suspension at 4°C, the immune complex was cleaned and magnetically separated. Then 200 µg spare protein, 5 µL PMSF, and 450 µL PBST were added to each IP group sample. Again, the immune complex was washed and magnetically separated after being shaken overnight at 4°C. The protein was diluted by 50 µL 1×SDS‐PAGE Sample Loading Buffer and denatured by heat. After centrifugation, supernatant was obtained and Western Blot was performed according to routine procedures.

### 5.8 Frozen Section and Immunofluorescence

Mice eyeballs were implanted with optimal cutting temperature compound (OCT, Sakura, Japan) and the corneas were made vertical. After the samples were completely solidified in liquid nitrogen, they were cut into 10‐µm‐thick slices along the coronal plane.

The frozen sections were fixed with 4% paraformaldehyde at room temperature for 10 min, and were blocked with 3% BSA at room temperature for 1 h after being washed with PBS. The BSA was removed, the GPX4 primary antibody prepared with 1% BSA was dripped onto the slices, and incubated in a wet box at 4°C overnight. After being washed with PBS, Alexa Fluor 488‐conjugated donkey anti‐rabbit IgG (#ab150077; Abcam, UK) incubated at room temperature for 2 h in the dark. Incubate with 4,6‐Diamidino‐2‐phenylindole (DAPI) for 5 min, cover and seal the frozen sections. Finally, οbserve them and photograph using Laser Scanning Confocal Microscopy (LSM880 with Airyscan; Zeiss, Germany).

### 5.9 TUNEL Analysis

Frozen sections were fixed with 4% paraformaldehyde for 30 min and permeabilized with 0.1% Triton X‐100 for 2 min at room temperature. According to the manufacturer's protocol, TUNEL working solution in In Situ Cell Death Detection Kit (Roche, Germany) was prepared and dropped on the frozen sections in the dark. Then these sections were incubated in a wet box at 37°C for 1 h. After PBS washing, the sections were labeled with DAPI and then sealed. Immediately observe and photograph under Laser Scanning Confocal Microscopy (LSM880 with Airyscan; Zeiss, Germany).

### 5.10 Measurement of ROS in Tissue

Reactive oxygen species (ROS) in tissues were detected with CM‐H2DCFDA probe (Invitrogen, USA). GENMED working solution was prepared according to the manufacturer's protocol. At room temperature, pre‐cooled cleaning solution was added to the frozen slices and removed after a few moments. Proper amount of working solution was added to the slices which were then incubated in a moist incubator at 37°C for 20 min. Remove the working solution from the slices, add the cleaning solution, and remove it after a few moments. DAPI were added before the slices being sealed. Observe them and photograph using Laser Scanning Confocal Microscopy (LSM880 with Airyscan; Zeiss, Germany).

### 5.11 Measurement of Lipid Peroxidation

For the cells inoculated in confocal microdishes, HBSS diluted into 2 µm C11 BODIPY 581/591 (ABclonal, USA), an oxidation‐sensitive fluorescent lipid peroxidation probe was used to incubate the cells for 60 min at 37°C in the dark. After being washed with sterile PBS for 3 times, confocal microscopy (LSM880 with Airyscan; Zeiss, Germany) was performed directly, and fluorescence at 510–550 nm and larger than 580 nm was detected by using excitations with 488 and 565 nm excitators. For frozen sections, C11 BODIPY probe diluted with PBS at 2 µm concentration was incubated at 37°C for 20 min in the dark, and then washed with PBS 3 times for 5 min each time. Confocal microscopy imaging was performed after DAPI added to the sections and sections sealed.

For flow cytometry, the adherent cells were incubated with C11 BODIPY probe and cleaned with PBS three times. After digestion with pancreatic enzymes, the cells were re‐suspended in PBS containing 5% FBS. Flow cytometer (Attune NxT; Thermo, USA) was used to detect cell reduction levels by measuring signals greater than 580 nm in the FL2 channel excited by the 565 nm laser.

### 5.12 Measurement of GSH

GSH was detected by GSSG/GSH Quantification Kit (Dojindo, Japan) according to the manufacturer's protocol. In brief, it is to prepare various working solutions first. Then the cells were treated with HCl, SSA, and other reagents, and then 40 µL GSSG Standard Solution, GSH Standard Solution, GSSG sample or GSH sample were added to each hole of the 96‐well plate, and 60 µL Buffer Solution was added before being cultured at 37°C for 1 h. Finally, 60 µL Substrate Working Solution and 60 µL Enzyme/Coenzyme Working Solution was added successively and cultured at 37°C for 10 min. Absorbance was measured at 405 nm by the microplate reader (MD190; MolecularDevices, USA), and GSSG concentration and total glutathione concentration were converted by corresponding standard curve. GSH = Total Glutathione (GSH + GSSG)—GSSG×2.

### 5.13 Measurement of MDA

MDA was detected by MDA Assay Kit (Dojindo, Japan) according to the manufacturer's protocol. In brief, it is to prepare various working solutions first. Next, after centrifugation, cleaning and other procedures, the cell samples were added with 100 µL Antioxidant PBS solution and 100 µL Lysis Buffer. After mixing and standing, 250 µL Working solution was added into each microtubule and the samples were cultured at 95°C for 15 min. The samples were cooled in the ice bath for 5 min, and then 100 µL supernatant was added to the 96‐well black plate after centrifugation. Finally, the fluorescence intensity was measured by multifunctional enzyme marker (Spectra Max M5; Molecular Devices, USA). The MDA concentration in the samples were calculated by the standard MDA curve.

### 5.14 Measurement of Intracellular Fe^2+^

The cells were inoculated in confocal microplates according to the manufacturer's protocol. After cell adhesion, Ferro Orange (Dojindo, Japan), a 2 µm ferrobivalent iron ion probe diluted with DMEM/F12, was incubated in the cell incubator for 1 h in the dark, and then washed with sterile PBS for 3 times. Using confocal microscopy (LSM880 with Airyscan; Zeiss, Germany) imaging, detection of laser intensity at 570–620 nm wavelength after excitation with 561 exciter.

### 5.15 Observation of Mitochondrial Morphology

Our team has already constructed the CMV‐COX8‐GFP vector lentivirus with the inner mitochondrial membrane protein COX8 and successfully transfected it into HCECs in the past, and the metastable cell line was amplified. Finally using confocal microscopy (LSM880 with Airyscan; Zeiss, Germany) imaging to detect cell mitochondrial morphology by capturing cell GFP green fluorescence signals.

### 5.16 Statistical Analysis

GraphPad Prism 9 (GraphPad Software, USA) was used to make statistics on the data in this study. The experimental results were repeated more than three times. Experimental data were presented in the form of mean ± SEM. Analysis of Variance (ANOVA) and Mann‐Whitney U test were used to evaluate the differences of parameters’ mean between groups. *p* < 0.05 was considered to be statistically significant.

## Author Contributions

L.L. and Z.L. designed the study and contributed to writing the manuscript. Z.L., X.Y., and J.L. conducted the majority of the experiments and analyzed the data. Z.W., J.L., and C.D. participated in the animal experiments. X.M., W.C., Q.Z., and others engaged in significant scientific discussions, oversaw the research, provided valuable insights, reviewed and commented on the manuscript. All authors contributed to the article and approved the submitted version.

## Conflicts of Interest

The authors declare no conflicts of interest.

## Supporting information




**Supporting File**: advs74548‐sup‐0001‐SuppMat.docx.

## Data Availability

The data that support the findings of this study are available from the corresponding author upon reasonable request.
