# Supplementary material for: SIRT1 Prevents Ferroptosis in Corneal Epithelial Cells by Enhancing HIF1α Protein Stability in Dry Eye Disease
Source: Adv Sci (Weinh). 2026 Feb 25;13(26):e22806. doi: 10.1002/advs.202522806 (PMC13159161; doi:10.1002/advs.202522806)
Supplement: Supplementary file 1 — Supporting File: advs74548‐sup‐0001‐SuppMat.docx. [file ADVS-13-e22806-s001.docx]

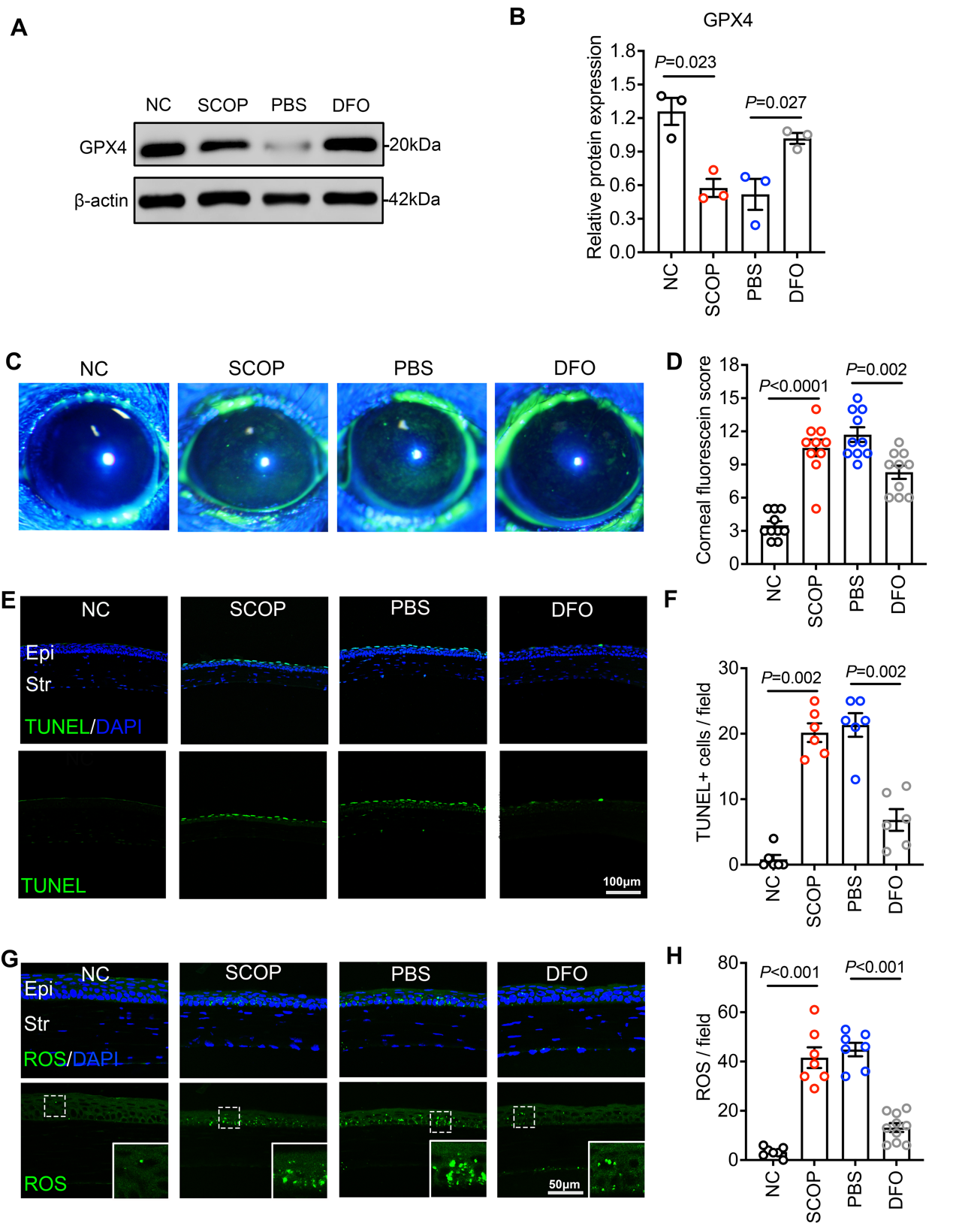


**Figure S1.** The ferroptosis inhibitor DFO alleviates ferroptosis in corneal epithelial cells in dry eye mice. Mice in the dry eye group were only injected subcutaneously with scopolamine hydrobromide (SCOP), while those in the treatment groups received PBS or DFO eye drops. A, B) WB analysis of GPX4 expression in cornea obtained from the negative control (n=6), with SCOP injected alone (n=3), with PBS (n=3), with DFO (n=3) eye drops, respectively for 5 days. C, D) Representative images of fluorescence staining for mice cornea from the negative control (n=10), with SCOP injected alone (n=10), with PBS (n=10), with DFO (n=10) eye drops, respectively for 5 days. Quantification of the corneal staining score of mice under the slit-lamp as shown. E, F) Representative fluorescence image and quantitative data for TUNEL apoptosis assay to detect apoptotic corneal epithelial cell for mice cornea from the negative control(n=6), with SCOP injected alone(n=6), with PBS(n=6), with DFO(n=6) eye drops, respectively for 5 days. Epi: epithelium; Str: stroma. Scale bar: 100μm. (G, H) CM-H2DCFDA fluorescent probe was used to mark the intracellular ROS accumulation and ROS count statistics in frozen slices of mouse cornea from the negative control (n=5), with SCOP injected alone (n=7), with PBS (n=7), with DFO (n=10) eye drops, respectively for 5 days. Epi: epithelium; Str: stroma. Scale bar: 50μm. NC, negative control; SCOP, scopolamine hydrobromide. Epi, corneal epithelium; Str, Corneal stroma. Data are presented as mean ± SEM from at least three independent experiments. Statistical significance was determined as indicated.


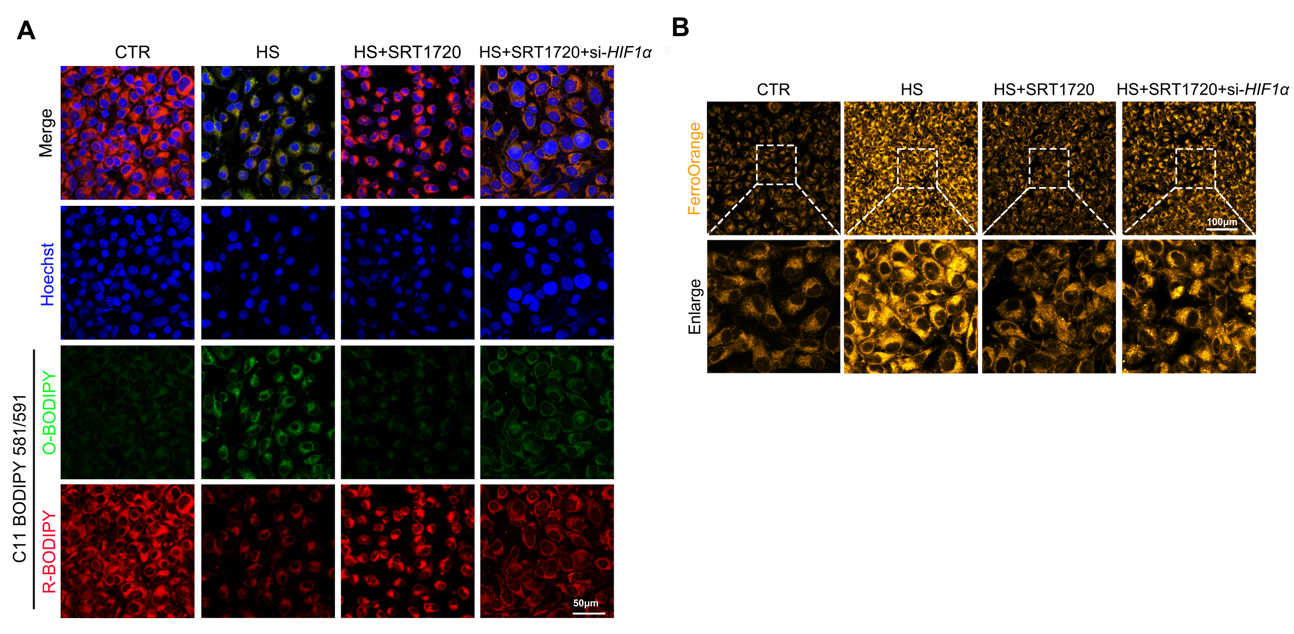


**Figure S2.** Knockdown of HIF1α abrogates the protective effect of SRT1720 against hyperosmosis-induced ferroptosis in HCECs. A) Representative fluorescent images of HCECs stained with C11 BODIPY 581/591 (red: reduced form,591 nm; green: oxidized form, 510 nm) under the following conditions for 72 hours: control group (CTR, 310 mOsm), hyperosmotic group (HS, 450 mOsm), HS supplemented with SRT1720 (1 μM), and HS with SRT1720 following HIF1α knockdown (si-*HIF1α*). Nuclei were stained with Hoechst (blue). Scale bar = 50 μm. B) Representative fluorescence microscopy imaging results of FerroOrange probe to detect intracellular Fe2+ content in HCECs. Scale bar: 100 μm.

Table S1. siRNA sequences were as follows:

| siRNA | Sequence（5’-3’） |
| --- | --- |
| si-*NC*-F | UUCUCCGAACGUGUCACGUTT |
| si-*NC*-R | ACGUGACACGUUCGGAGAATT |
| si-*SIRT1*-1-F | GUGGCAGAUUGUUAUUAAUTT |
| si-*SIRT1*-1-R | AUUAAUAACAAUCUGCCACTT |
| si-*SIRT1*-2-F | CGGGAAUCCAAAGGAUAAUTT |
| si-*SIRT1*-2-R | AUUAUCCUUUGGAUUCCCGTT |
| si-*HIF1α*-1-F | GUUGCCACUUCCACAUAAUTT |
| si-*HIF1α*-1-R | AUUAUGUGGAAGUGGCAACTT |
| si-*HIF1α*-2-F | CCGUAUGGAAGACAUUAAATT |
| si-*HIF1α*-2-R | UUUAAUGUCUUCCAUACGGTT |

Table S2. The related primers used in RT-qPCR were as follows:

| Gene | Sequence（5’-3’） |
| --- | --- |
| Human *β-actin*-F | TCATGAAGTGTGACGTGGACATC |
| Human *β-actin*-R | CAGGAGGAGCAATGATCTTGATCT |
| Human *GPX4*-F | GAGGCAAGACCGAAGTAAACTAC |
| Human *GPX4*-R | CCGAACTGGTTACACGGGAA |
| Human *SIRT1*-F | TAGCCTTGTCAGATAAGGAAGGA |
| Human *SIRT1*-R  Human *ACLS4*-F  Human *ACSL4*-R  Human *NCOA4*-F  Human *NCOA4*-R | ACAGCTTCACAGTCAACTTTGT  TCTGCTTCTGCTGCCCAATT  CGCCTTCTTGCCAGTCTTTT  CTATATCCAGGTGCCAGAGCAG  TTGCTTACAAGAAGCCACTCAC |
| Human *HIF1α*-F | AGCCGAGGAAGAACTATG |
| Human *HIF1α*-R | ACTGAGGTTGGTTACTGTT |
| Mouse *β-actin*-F | CAGCCTTCCTTCTTGGGTATG |
| Mouse *β-actin*-R | GGCATAGAGGTCTTTACGGATG |
| Mouse *Gpx4*-F | CAGGAGCCAGGAAGTAAT |
| Mouse *Gpx4*-R | CAGCCGTTCTTATCAATGAG |
| Mouse *Sirt1*-F | CCTGACTTCAGATCAAGAGACGGTA |
| Mouse *Sirt1*-R  Mouse *Acsl4*-F  Mouse *Acsl4*-R  Mouse *Ncoa4*-F  Mouse *Ncoa4*-R | CTGATTAAAAATGTCTCCACGAACAG  CCACACTTATGGCCGCTGTT  GGGCGTCATAGCCTTTCTTG  GCGAATTCATGAACACATCCCTG  GCGAATTCTCACATCTGTAGAGG |
| Mouse *Hif1α*-F | TAAGGCATCAGCATACAGT |
| Mouse *Hif1α*-R | AAGTGGCAGACAGGTTAA |
